# Exploring the rhizospheric bacterial community of selected millets for plant growth promotion activity in tomato

**DOI:** 10.3389/fpls.2025.1629184

**Published:** 2025-09-26

**Authors:** Anitha Sakthivel, Balasubramanian Santhanalakshmi, Jesudass Joseph Sahayarayan, Ganeshan Sivanandhan, Ravishankar Ram Mani, Soon Woong Chang, Balasubramani Ravindran, Santosh Chokkakula, Gnanajothi Kapildev

**Affiliations:** ^1^ Translational Plant Research Laboratory, Department of Microbial Biotechnology, Bharathiar University, Coimbatore, Tamil Nadu, India; ^2^ Department of Bioinformatics, Alagappa University, Karaikudi, Tamil Nadu, India; ^3^ Centre for Research, Dhanalakshmi Srinivasan University, Tiruchirappalli, Tamil Nadu, India; ^4^ Department of Pharmaceutical Biology, Faculty of Pharmaceutical Sciences, UCSI University, Kuala Lumpur, Malaysia; ^5^ Department of Civil & Energy System Engineering, Kyonggi University, Suwon, Gyeonggi-Do, Republic of Korea; ^6^ Department of Microbiology, Faculty of Arts Science Commerce and Management, Karpagam Academy of Higher Education, Coimbatore, Tamil Nadu, India; ^7^ Department of Microbiology, Chungbuk National University Medical College, Cheongju, Republic of Korea

**Keywords:** antagonistic activity, millet, PGPR, plant growth promotion, rhizosphere

## Abstract

Millets are highly nutritious crops mostly cultivated in xeric regions of Asia and Africa. The specific agro-climatic characteristics of millets enable the rhizosphere to host diverse microorganisms that assist in the crop’s progression under harsh weather conditions. This research work intends to evaluate the plant growth-promoting (PGP) potential of the rhizospheric microbes isolated from the soil of *Panicum sumatrense* and *Eleusine coracana* from Kunnanchala and Varagampadi, residing areas of Irulas Tribal Community located in the Attapadi Hills of Kerala and Tamil Nadu, respectively. A total of 53 bacterial isolates with unique colony morphology were initially subjected to the nitrogen fixation test. Twenty-six isolates that demonstrated positive results for nitrogen fixation were characterized for phosphate solubilization, ammonia, indole acetic acid (IAA), siderophore, hydrogen cyanide (HCN), and hydrolytic enzyme production, molecular characterization, and antagonistic activity against the common plant pathogens *Fusarium oxysporum* and *Colletotrichum gloeosporioides*. The principal component analysis revealed that SA1 (*Staphylococcus gallinarum*) and MS6 (*Kosakonia sacchari*) exhibited the highest values for IAA production (0.37 ± 0.015 mg/mL) and PSI (3.36 ± 0.03), respectively. MS3 (*Bacillus velezensis*) demonstrated the most promising results in antagonism (91.32 ± 0.57) and ammonia production (0.11 ± 0.020 mg/mL). All the bacterial isolates exhibited a notable improvement in germination, shoot length, root length, and vigor index of *Solanum lycopersicum*. The most prominent results in germination studies were noted in K. sacchari (MS6), with the most pronounced effects, including a 100% germination rate, 2.58 ± 0.01 cm of shoot length, 7.61 ± 0.03 cm of root length, and a vigor index of 1019. The results of the *invitro* PGP traits are sufficient to support future tests on the promotion of growth *invivo* for these seven strains in a single or consortium.

## Introduction

Millet is a small seeded cereal crop from the family Poaceae, classified under the C4 grasses, and usually cultivated in xeric and semi-xeric regions (Han et al., 2017). Major and minor millets are two categories of millets which include *Eleusine coracan* (finger millet), *Panicum miliaceum* (proso millet)*, Setaria italica (*foxtail millet), *Pennisetum glaucum* (pearl millet), *Echinochlo acolona* (barnyard millet), *Digitaria bursa* (black fonio millet), *Digitaria exilis* (white fonio millet), *Panicum miliare* (little millet)*, Paspalum scrobiculatum* (Kodo millet), *Eragrostis tef (*teff millet), respectively (Annor et al., 2014). Millets are known for their potential to withstand harsh, dry environments and challenging environments, including changing climates and nutrient-poor soil (Sharma and Ortiz, 2000). These characteristic features make it a wise alternative cereal crop for regions with limited rainfall, irrigation, and nutrient-poor soil. Millets typically flourish in higher temperatures and can reproduce with minimal water input, making them thermophilic and xerophilic (Saxena et al., 2018). Together with their nutritional benefits, millets offer short-term cultivation periods, reduced water requirements, and adaptability to adverse climatic conditions. When compared to major cereal crops, millets yield high on marginal land. These specific agro-climatic characteristics render millets particularly well-suited for cultivation in semi-arid regions of Asia and Africa, where other major crops frequently encounter challenges (Belton and Taylor, 2004).

The rhizosphere is an area of the soil that harbors microorganisms with a significant connection to plant physiology, involving complex organic material decomposition, resistance to stresses (both biotic and abiotic), and contributing to plant development. The resilience of millets is largely driven by the microbial community present in the rhizosphere. The term “plant growth-promoting microorganisms” (PGPM) encompasses a diverse array of organisms that reside in the rhizosphere, which includes plant growth-promoting rhizobacteria (PGPR), antagonistic microorganisms, beneficial symbionts, mycorrhizal fungi, mycoparasitic fungi, endophytic bacteria, and fungi (Meena et al., 2017). The PGPR present in the rhizosphere region boosts plant growth and development by engaging in various positive mechanisms. These include increasing the nutrient uptake, fixing the atmospheric nitrogen, suppressing the growth of phytopathogens (Wang et al., 2022; Sheteiwy et al., 2023), and improving the host plant resistance against various abiotic and biotic stressors (Selvakumar et al., 2012; Zolla et al., 2013) and results in increased plant growth and yield (Berg, 2009; Huang et al., 2014).

The principal role of PGPR in nurturing plant growth is achieved through two distinct mechanisms: direct and indirect (Kong and Liu, 2022). The direct mechanisms involve the synthesis of plant growth hormones such as cytokinin, gibberellins, and auxins, together with the solubilization of nutrients, including phosphate. The indirect mechanisms include the production of antibiosis to combat phytopathogens and ACC deaminase to resist various stressors (Barea et al., 2005). *Azotobacter, Azospirillum, Acinetobacter, Azoarcus, Arthrobacter, Burkholderia, Bacillus, Beijerinckia, Enterobacter, Erwinia, Klebsiella, Gluconacetobacter, and Serratia*. Some of the most extensively studied PGPR for plant growth promotion (PGP) belong to the genus *Rhizobium* and *Pseudomonas* (Murphy et al., 2003; Esitken et al., 2006). *Acinetobacteria* have been shown to enhance the yields of various crops, particularly those of global importance like rice, maize, and wheat (Mahmoud et al., 2024).

To address the growing food demands of an increasing population, agrochemicals use has escalated to boost crop growth and productivity. These agrochemicals are pivotal in increasing crop productivity. However, their prolonged usage may lead to soil infertility through processes such as diminishment of soil organic matter, nitrogen leaching, depletion of soil carbon, and soil compaction. To overcome the usage of hazardous chemicals in agriculture, a wise alternative is the application of biofertilizers derived from PGPR (Nader et al., 2024), while endophytes (Mahmoud et al., 2025) can also act as promising agents that enhance plant growth and support eco-friendly, sustainable agriculture. It is an environmentally friendly and cost-effective alternative that can enhance soil fertility and pave the way for sustainable agriculture. This research work intends to isolate, characterize, and identify PGPRs that possess PGP properties from the region’s millet rhizosphere.

## Materials and methods

### Sample collection

**Figure 1:**
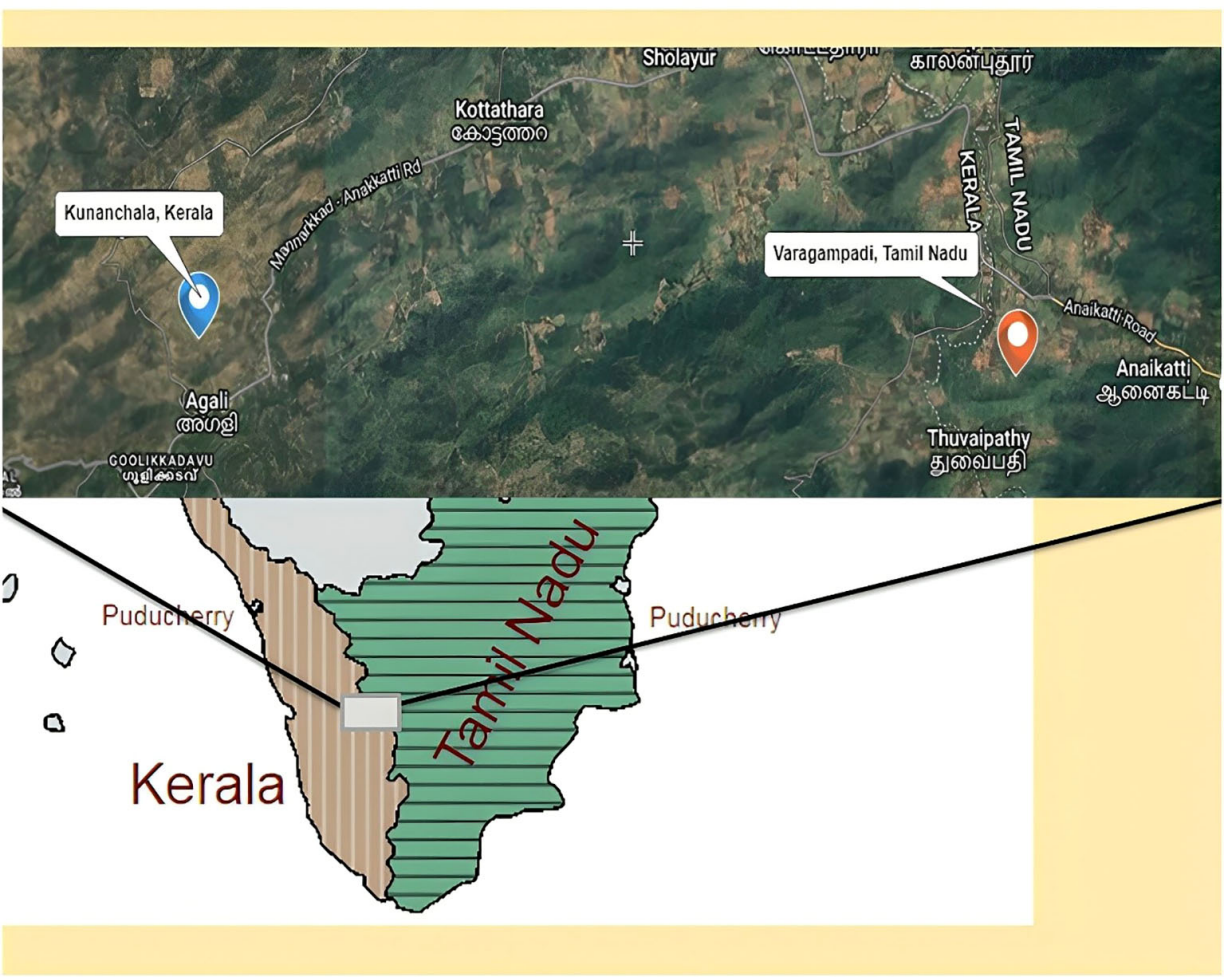
Rhizospheric soil sample collection area Kunnanchala (Lat 11.100603°, Long 76.609637°) and Varagampadi (Lat 11.069483°, Long 76.710132°), located in the Attapadi Hills of Kerala and Tamil Nadu, respectively.

Rhizospheric soil samples from 2 different types of millets, at a depth of 6–10 cm, were collected: two from Kunnanchala (5 soil samples/*Panicum sumatrense* and *5* soil samples/*Eleusine coracana*), and one from Varagampadi (5 samples/*P. sumatrense* – wild type), a region of the Irulas tribal community located in the Attapadi Hills of Kerala and Tamil Nadu, respectively (**Figure 1**). The soil samples were meticulously collected in aseptic containers and immediately transported and processed in the laboratory under sterile conditions.

### Bacterial isolation from rhizospheric soil

The rhizospheric soil samples were obtained, transported, and shade-dried to eliminate moisture content. Thereafter, the sample was subjected to serial dilution ranging from 10^-1^to10^-10,^and each dilution was plated on the nutrient agar medium (Peptone 5 g/L, Sodium chloride 5 g/L, HM peptone B 1.5 g/L, Yeast extract 1.5 g/L, Agar 15 g/L, Final pH (at 25°C) 7.4 ± 0.2) for bacterial isolation after incubating for 2–3 days at 37°C. During this period, the colonies on the plates were compared with control plates to ensure the absence of contamination. The colonies were randomly chosen based on their distinct morphological features. Each colony was then subjected to a process of pure culture isolation. The obtained pure cultures were named with unique number codes and preserved in glycerol stocks, which were stored at a temperature of –20°C for future use.

### Nitrogen fixation test

Qualitative confirmation of nitrogen fixation was achieved through the use of Jensen’s Nitrogen-free bacteria medium (JNFb) in which the inoculated pure bacterial cultures were kept under incubation at 28 ± 2°C for 4 days. The growth of bacteria on the JNFb medium indicates a positive result and qualitative confirmation for nitrogen fixation. The isolates were evaluated for their capacity to fix atmospheric nitrogen in the soil on this medium (Andy et al., 2020).

#### Invitro analysis of PGP activities

The isolates’ qualitative and quantitative assessment of Indole-3-acetic acid (IAA) synthesis was analyzed as per the protocol followed by Goswami et al. (2014). Based on the method followed by Cappuccino and Sherman (1992), the ammonia production was quantified by the calorimetric method. Phosphate solubilization by the isolates was analyzed by Pikovskaya medium (Pikovskaya, 1948). The halo zone formation signified phosphate solubilization (Mehta and Nautiyal, 2001) and it was quantified using the phosphate solubilization index (PSI), Goswami and Deka, 2020). Additionally, HCN and siderophore production were determined as per the protocol by Castric (1975) and Alexander and Zuberer (1991), respectively.

### Hydrolytic extracellular enzyme activity

The chosen bacterial isolates were assessed for their capacity to produce catalase by adding 3% hydrogen peroxide drop by drop to a clean glass slide containing a 48-hour bacterial culture. The presence of effervescence showed a positive result for catalase activity (Kumari et al., 2018). An *invitro* plate assay was conducted to detect the hydrolytic extracellular enzyme production. The protease production was screened by spot-inoculating the isolated strains on a plate containing skim milk agar and incubate at 30°C for 24 to 48 hours. After incubation, the generation of a clear halo zone circle the colony indicates a positive result for the degradation of protease.

The isolates were dot-inoculated on starch agar medium and incubated for 24 to 48 hours at 30°C to determine the amylase activity. Subsequently, the plates were saturated with iodine solution, left for one minute, and discarding the excess iodine. The formation of a colorless zone surrounding the colony signifies the production of the amylase enzyme. The cellulase production was screened by inoculating the strains in carboxy methyl cellulose Congo red agar medium and incubating at 28 ± 2°C for 2–3 days. Following incubation, the formation of a halo zone indicates a positive result for the synthesis of the cellulase enzyme. For the synthesis of lipase enzyme, the isolated strains were spot-inoculated on a tributyrin agar medium and kept under incubation for 2 days at 37°C. The clear zone formed around the colony showed the ability of the isolated strains to produce lipase enzymes (Sachan et al., 2018).

### *In vitro* screening of antifungal activity

The isolated strains were evaluated for their *invitro* antifungal characteristics against the fungal phytopathogens *Fusarium oxysporum* and *Colletotrichum gloeosporioides* using a dual culture technique with a potato dextrose agar (PDA) medium. The fully grown active culture of phytopathogens was prepared by cutting 5mm diameter mycelial disks and placing them at the center of fresh sterile PDA plates. The overnight-grown isolated strains were then streaked uniformly around the mycelial discs served as the control. The plates were kept under incubation at 26°C for 7 days. Following incubation, the plates were examined for the zone of inhibition against the fungal phytopathogens. All experiments were conducted in triplicate. The rate of inhibition can be calculated by the formula.


$$IR(\%)\;=\frac{C-T}{C}\times 100$$

IR: inhibition rate; T: Diameter of the fungal colony in the antagonist plate, C: Diameter of the fungal colony in the control plates; (Ezrari et al., 2021).

### Molecular characterization of potent PGPR

Genomic DNA was extracted from the selected strains using the phenol/chloroform method as described by Kumari et al. (2018). The overnight-grown bacterial strains were centrifuged at 10,000 rpm for 10 minutes. Following centrifugation, the supernatant was cast off and 30 μL of 10% SDS and then 3 μL of proteinase K were mixed with the pellets, which were then incubated for 1hr at 37°C. Subsequently, the mixer mentioned above was subjected to another centrifugation process, during which phenol-chloroform-isoamyl alcohol (25:24:1) was added. This step precipitated genomic DNA with ice-cold ethanol. Following the extraction of genomic DNA from the selected strains, DNA amplification was conducted using two universal primers, 16S rRNA (16SF & 16SR), via polymerase chain reaction (PCR).

### Impact of bacterial isolates on seed germination

The selected seven bacterial strains were progressed for the seed germination study. The seeds of the *Solanum lycopersicum* PKM 1varietywere sourced from the Tamil Nadu Agricultural University, Coimbatore. Prior to the commencement of the study, the seeds were subjected to surface sterilization with 2% sodium hypochlorite for duration of 2 min. Thereafter, the seeds were rinsed 3 to 5 times with autoclaved distilled water to eliminate any residual surface disinfectant (Pandey and Gupta, 2020). The germination study was conducted as 8 treatments.

T1 - Seeds soaked in water (Control)

T2 - Seeds bioprimed with R4

T3 - Seeds bioprimed with R5

T4- Seeds bioprimed with S7

T5- Seeds bioprimed with SA1

T6- Seeds bioprimed with MS2

T7- Seeds bioprimed with MS3

T8- Seeds bioprimed with MS6

The surface-sterilized seeds were bioprimed with the seven bacterial isolates (10^8^ CFU mL^−1^) separately in a sterile tube for 1 hour at a 180-rpm orbital shaker. A control set of surface-sterilized seeds was prepared by soaking them in autoclaved distilled water. Subsequently, the bioprimed and control seeds were placed on a sterile Petri plate containing wetted sterile cotton. Each plate contains 10 seeds, with three replicates per treatment. The seeds were observed for seed germination over a period of 10 days. The seed germination percentage (G%) and seed vigor index (VI) were calculated using the following formula, as outlined by Fahsi et al. (2021).


$$G\;\%=\frac{No\;of\;seeds\;germinatied\;}{Total\;no.of\;seeds}\times 100$$

VI=(Mean of shoot length+Mean of root length)×G%

### Statistical analysis

The obtained data were assessed through one-way ANOVA in SPSS Statistics version 16.0 software. The significant variance among the treatment means was calculated using Duncan’s multiple range test at a *p* < 0.05 significance level. The experiments were expressed as the mean of triplicate ± standard error (SE). The Principal Component Analysis (PCA) was employed to investigate the correlation between the bacterial isolates and their PGP activity. The Bar graphs were plotted using Origin 2018–64 Bit software

## Results

### Isolation and characterization of bacterial isolates

In the present work, 53 morphologically distinct isolates were isolated from millets (*P. sumatrense* and *E. coracana*) rhizosphere soil using the spread plate technique. categorized based on their colony morphology. All the 53 isolates were assessed for their nitrogen fixation ability, and 26 were found to be capable of fixing nitrogen when inoculated in a nitrogen-free medium.

### *Invitro* PGP activity

**Figure 2:**
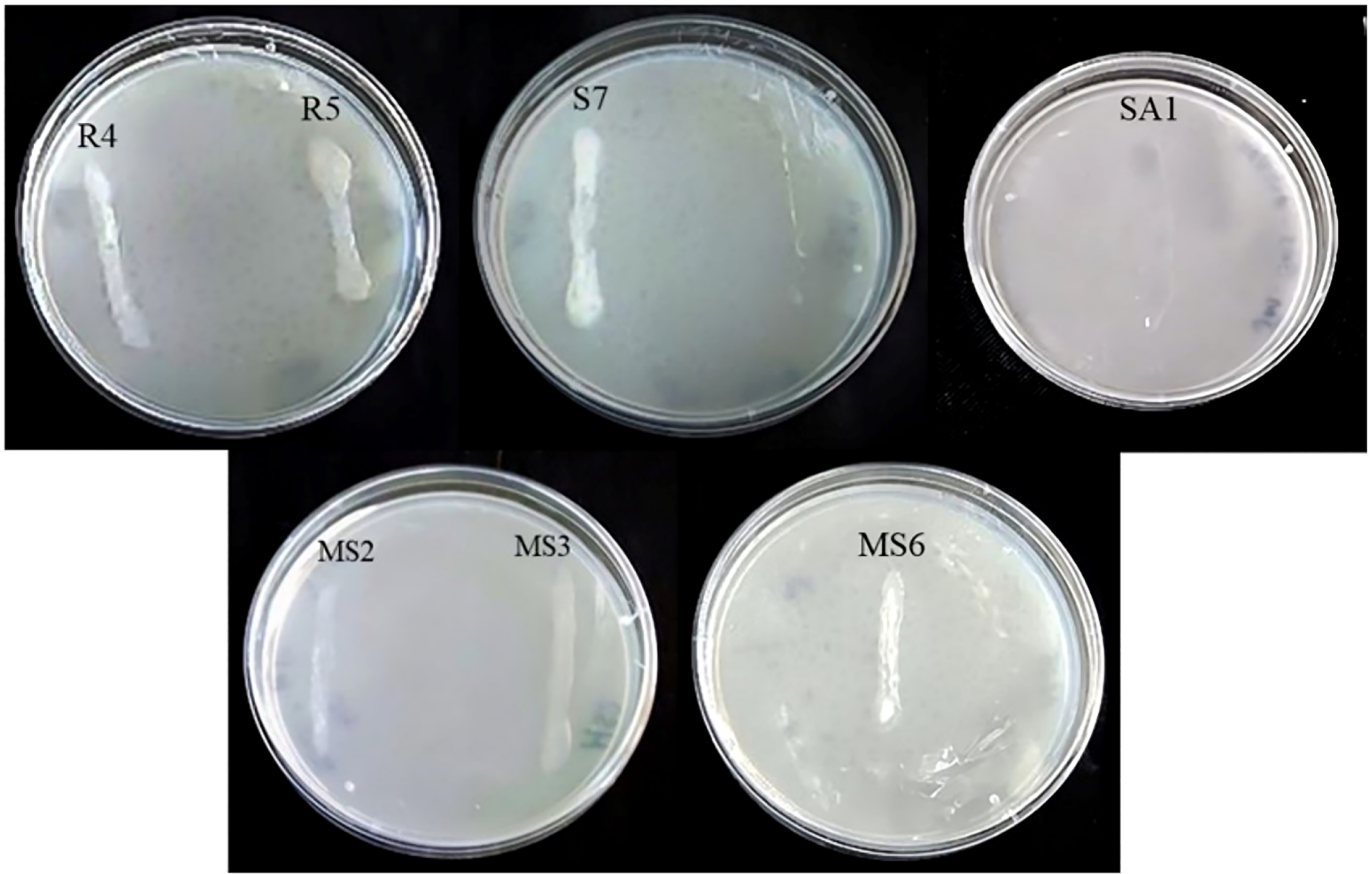
Bacterial Growth on Jenson’s medium.

*Invitro* assays revealed that, 14 isolates produced the phytohormone IAA, 23 isolates produced ammonia, 15 isolates were observed to solubilize phosphate, 3 isolates were identified to produce HCN, and 5 isolates were observed to produce siderophore. Based on the qualitative analysis of PGP properties, 7 of the 26 isolates (designated as R4, R5, S7, SA1, MS2, MS3, and MS6) (**Figure 2**) exhibited the most promising results for maximum PGP activities and were selected for further analysis of hydrolytic enzyme production, antagonistic activity, and germination assay.

**Figure 3:**
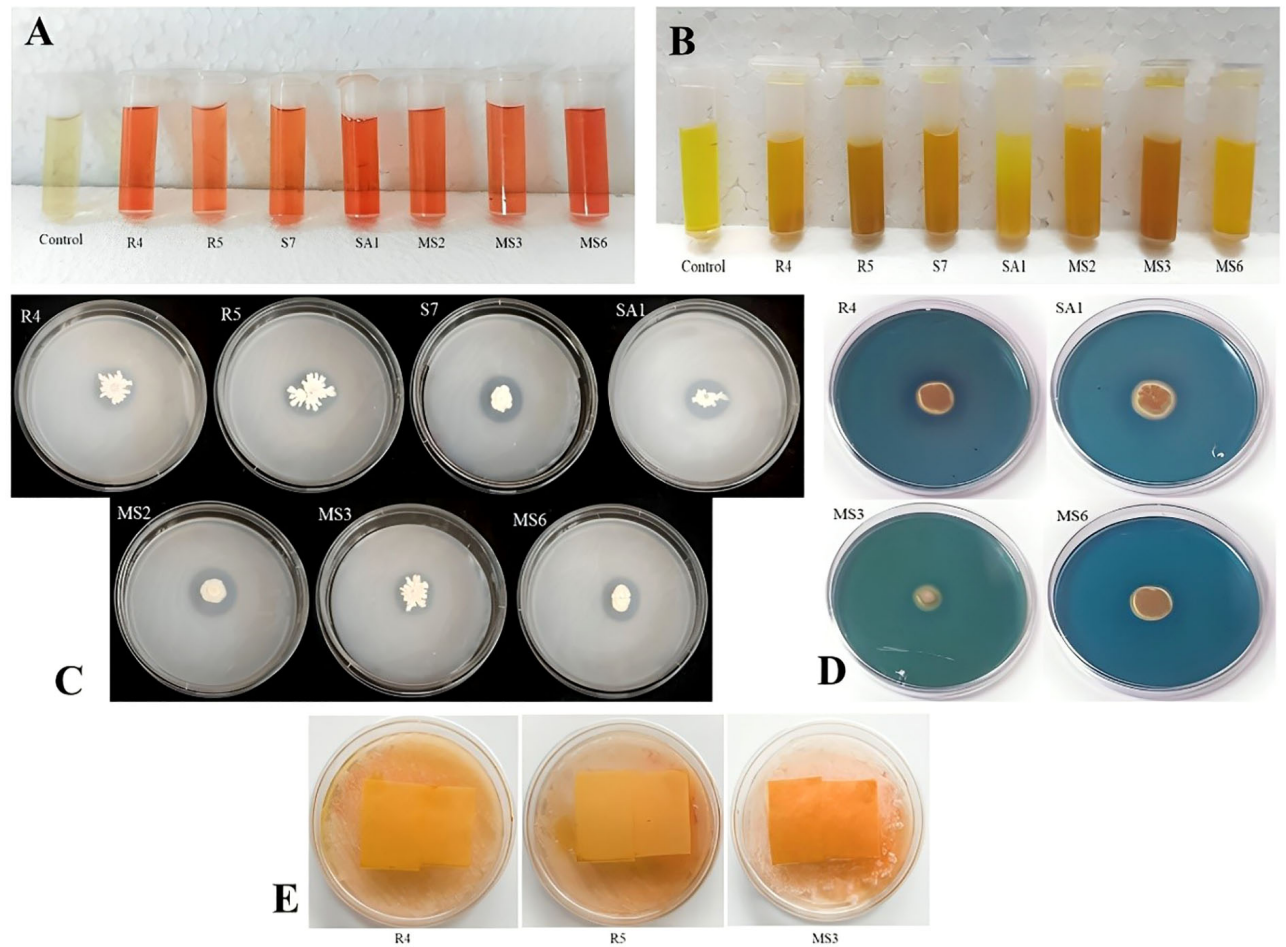
*Invitro* screening of the isolates for **(A)** IAA production, **(B)** Ammonia production, **(C)** phosphate solubilization activity **(D)** siderophore production, and **(E)** production of HCN.

**Figure 4:**
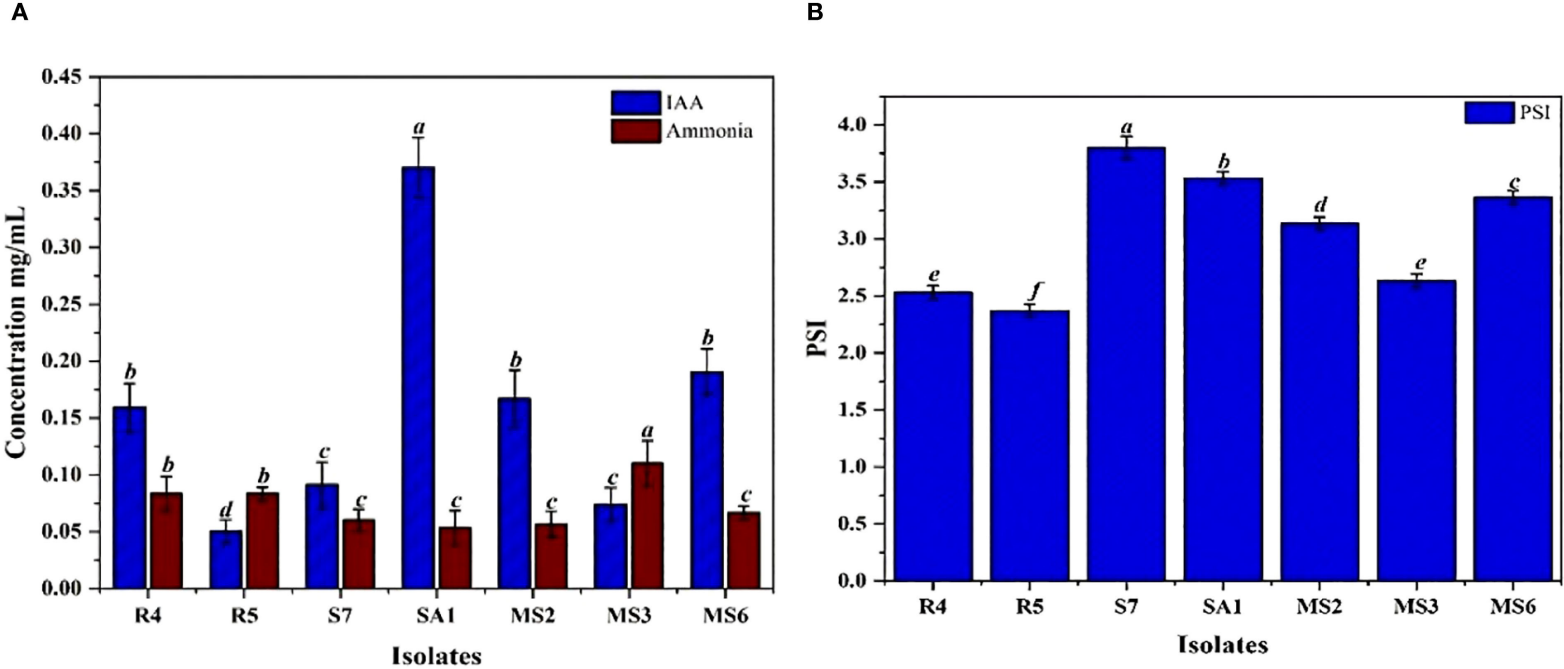
**(A)** Quantification of IAA and Ammonia production **(B)** phosphate solubilization index (PSI) by seven bacterial isolates, namely R4, R5, S7, SA1, MS2, MS3, and MS6. Bars with different letters show significant differences from each other (*p* < 0.05), and the liner bar shows the standard error.

The IAA production among the isolates varied from 0.05 to 0.37 mg/mL (**Table 1**). These results were quantified against a standard curve prepared from a precise concentration of standard IAA (**Figures 3A**, **4A**). The highest IAA production was observed in SA1, with a concentration of 0.37 ± 0.015 mg/mL, and the lowest was in R5 (0.05 ± 0.008 mg/mL For ammonia production, the isolates R4, R5, S7, SA1, MS2, MS3, and MS6 exhibited higher production ranging from 0.06 and 0.11 mg/mL (**Table 1**), (**Figures 3B**, **4A**). The highest level of ammonia was produced by MS3 with 0.11 ± 0.020 mg/mL. Upon investigating the phosphate solubilization ability of the isolates, a halo zone was observed around the bacterial colonies on Pikovskaya agar medium, with varying PSI values ranging from 2.36 to 3.80 (**Table 1**, **Figures 3C**, **4B**).

**Table 1:** *Invitro* screening of isolates for plant growth-promoting activities (qualitative and quantitative).

Bacterial isolates	*Invitro* assays (Qualitative and quantitative)					
	N2	IAA (mg/mL)	NH3 (mg/mL)	PSI	HCN	Siderophore
R4	+	0.18 ± 0.023	0.08± 0.008	2.53 ± 0.03	+	+
R5	+	0.05 ± 0.008	0.08± 0.003	2.36± 0.03	+	–
S7	+	0.09± 0.011	0.06± 0.008	3.80 ± 0.05	–	+
SA1	+	0.37 ± 0.015	0.06 ± 0.015	3.53± 0.03	–	+
MS2	+	0.17 ± 0.014	0.06 ± 0.006	3.13 ± 0.03	–	–
MS3	+	0.07 ± 0.008	0.11 ± 0.020	2.63 ± 0.03	+	+
MS6	+	0.20 ± 0.020	0.08 ± 0.015	3.36 ± 0.03	–	+

In the present work, the isolates, namely R4, R5, and MS3, demonstrated the capacity to produce HCN and the remaining were unable to shift the color of Whatman filter paper no. 1 (**Figure 3E**). The synthesis of siderophore by the isolates was confirmed by the generation of an orange halo zone surrounding the bacterial colony inoculated in the chrome azurol S (CAS) agar plates. The isolates R4, S7, SA1, MS3, and MS6 were found to be capable of producing siderophore, as evidenced by the results presented in **Figure 3D**.

### Hydrolytic enzyme activity

**Figure 5:**
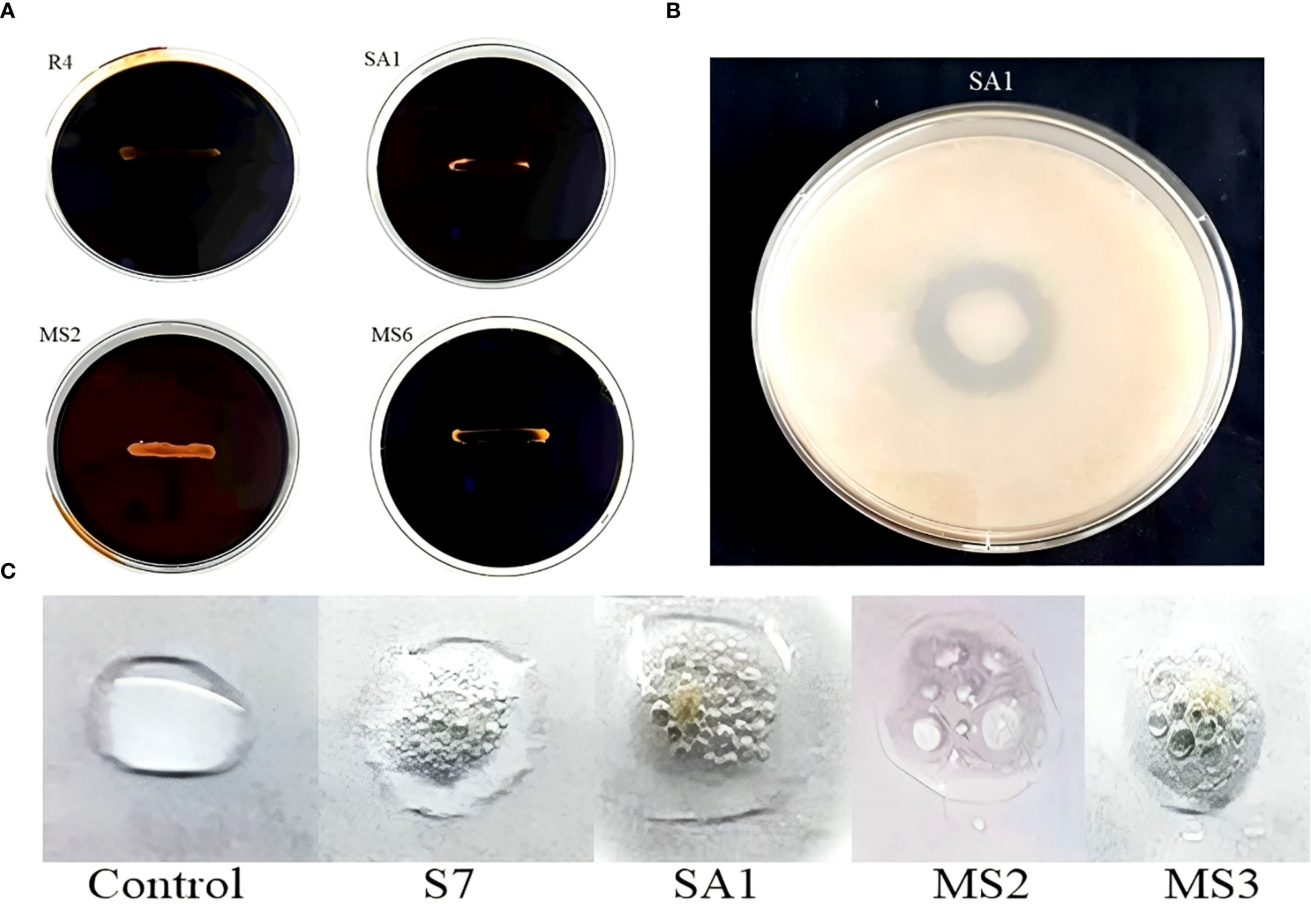
Enzyme activity of the isolates, confirmation of amylase **(A)** protease **(B)** and **(C)** catalase production.

From the conducted preliminary studies, seven best isolates were selected for the enzymatic studies. In the present work, four bacterial isolates, namely R4 (*E. hormaechei*), SA1 (*S. gallinarum*), MS2 (*B. amyloliquefaciens*), and MS6 (*K. sacchari*), produced amylase (**Figure 5A**). While only one bacterial isolate, SA1 (*S. gallinarum*) exhibited positive results for protease production. The clear zone formation circling the bacterial colony on skim milk agar showed a positive result for proteolytic enzyme production (**Table 2**; **Figure 5B**). The bacterial isolates S7 (*P. agglomerans*), SA1 (*S. gallinarum*), MS2 (*B. amyloliquefaciens*), and MS6 (*K. sacchari*) exhibited positive results for catalase production (**Table 2**; **Figure 5C**). No positive results were obtained for lipase production.

**Table 2:** Screening of isolates for the production of hydrolytic enzymes.

Bacterial isolates	Amylase	Protease	Lipase	Catalase
R4	+	–	–	–
R5	–	–	–	–
S7	–	–	–	+
SA1	+	+	–	+
MS2	+	–	–	+
MS3	–	–	–	+
MS6	+	–	–	–

### *Invitro* assessment of antifungal activity

**Figure 6:**
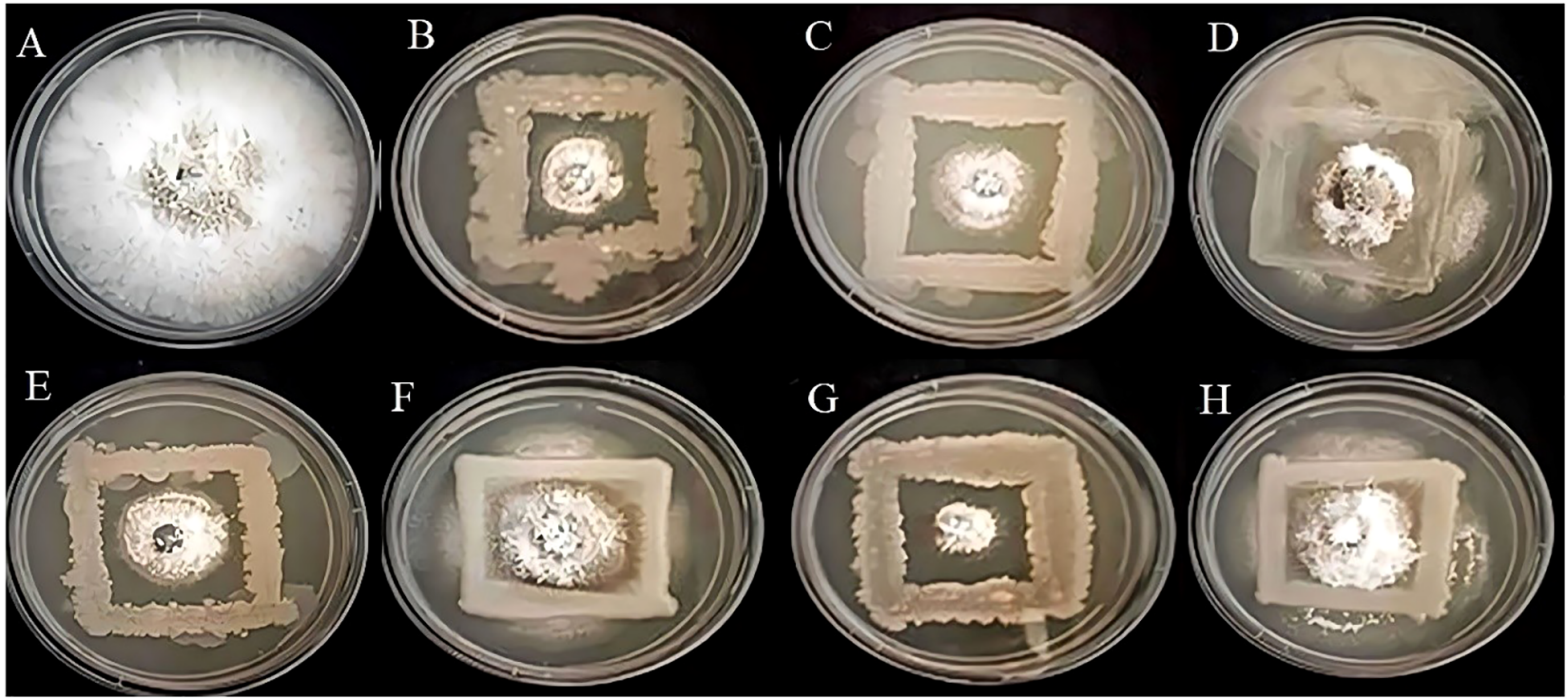
Antagonistic activity of *Fusarium oxysporum* **(A)** Control, **(B)** R4, **(C)** R5, **(D)** S7, **(E)** SA1, **(F)** MS2, **(G)** MS3, **(H)** MS6, respectively.

**Figure 7:**
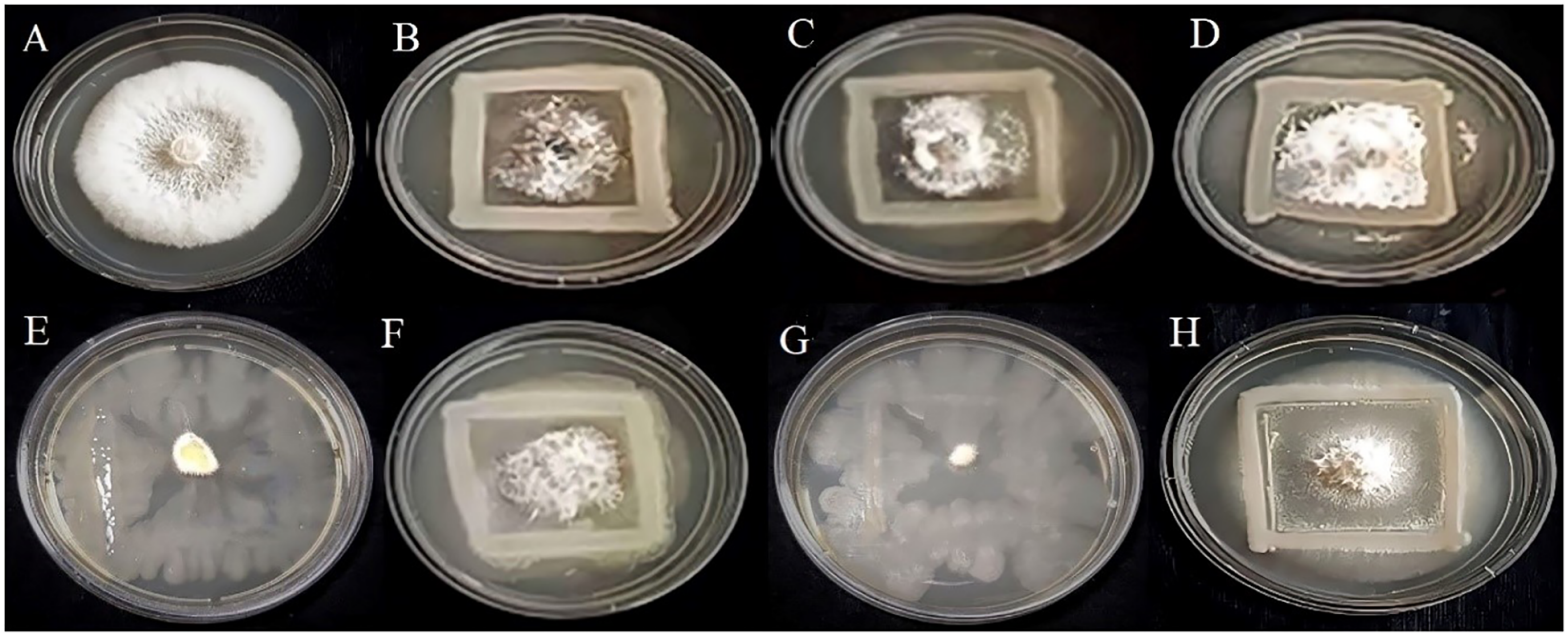
Antagonistic activity of *Colletotrichum gloeosporioides* **(A)** Control, **(B)** R4, **(C)** R5, **(D)** S7, **(E)** SA1, **(F)** MS2, **(G)** *MS3*, **(H)** MS6, respectively.

**Figure 8:**
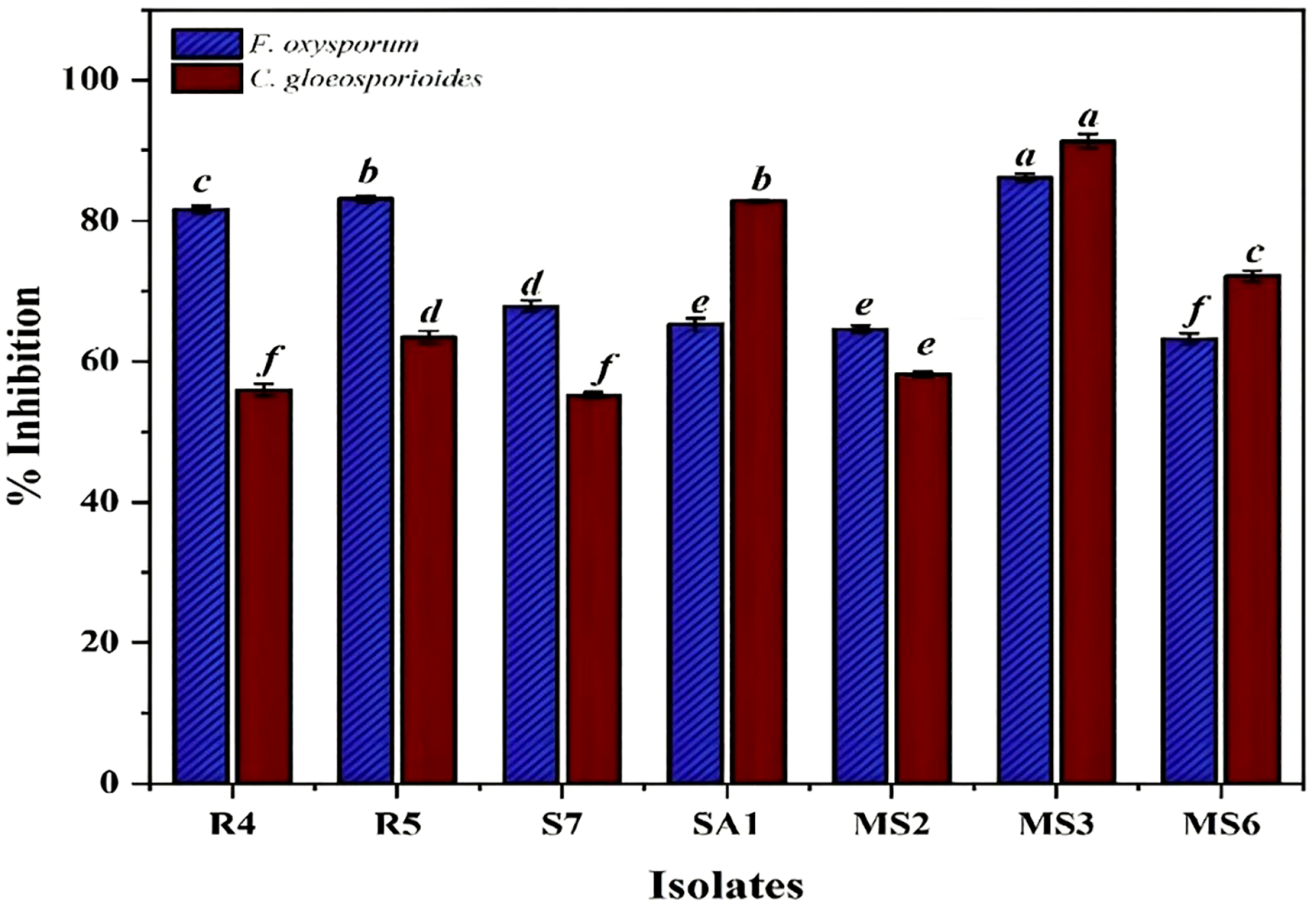
*Invitro* antagonistic activity by the seven bacterial isolates, namely R4, R5, S7, SA1, MS2, MS3, and MS6, against the phytopathogens *F.oxysporum* and *C. gloeosporioide.* Bars with different letters show significant differences from each other (*p* < 0.05) and the liner bar shows the standard error.

In the present study, based on the dual culture technique, all 7 bacterial isolates demonstrated the capacity to suppress the mycelial growth of the plant pathogens *F.oxysporum* and *C.gloeosporioides*, with varying degrees of efficacy. These two phytopathogens cause Fusarium wilt, Fusarium crown, and root rot (McGovern, 2015) and anthracnose (Lassois and de Bellaire, 2014) in crop plants. The antifungal activity of the bacterial strains R4, R5, S7, SA1, MS2, MS3, and MS6 exhibited a range of percent inhibition values against the pathogens, with values ranging from 63.19% to 86.15% for *F. oxysporum* and 55.18% to 91.32%for *C. Gloeosporioides* (**Figures 6**, **7**). The highest antagonism was observed in MS3 (*B. velezensis*) against *F. oxysporum* with an inhibition rate of 86.15%, followed by R5 (*B. siamensis*) 83.08%, R4 (*E. hormaechei*) 81.59%, S7 (*P. agglomerans*) 67.83%, SA1 (*S. gallinarum*) 65.22%, MS2 (*B. amyloliquefaciens*) 64.58%, and MS6 (*K. sacchari*) 63.19%.The antagonistic activity of *C.gloeosporioides* was more pronounced in MS3 (*B. velezensis*) 91.32%, SA1 (*S. gallinarum*) 82.78%, MS6 (*K. sacchari*) 72.12%, R5 (*B. siamensis*) 63.42%, MS2 (*B. amyloliquefaciens*) 58.15%, R4 (*E. hormaechei*) 55.93%, and S7 (*P. agglomerans*) 55.18% (**Table 3**; **Figure 8**).

**Table 3:** Antagonistic activity of the isolates against the phytopathogens.

Phytopathogens	R4	R5	S7	SA1	MS2	MS3	MS6
*Fusarium oxysporum*	81.59 ± 0.30	83.08± 0.28	67.83± 0.46	65.22± 0.51	64.58± 0.30	86.15± 0.31	63.19± 0.44
*Colletotrichum gloeosporioides*	55.93± 0.49	63.42± 0.50	55.18± 0.23	82.78± 0.14	58.15± 0.20	91.32± 0.57	72.12± 0.43

### Molecular identification

**Figure 9:**
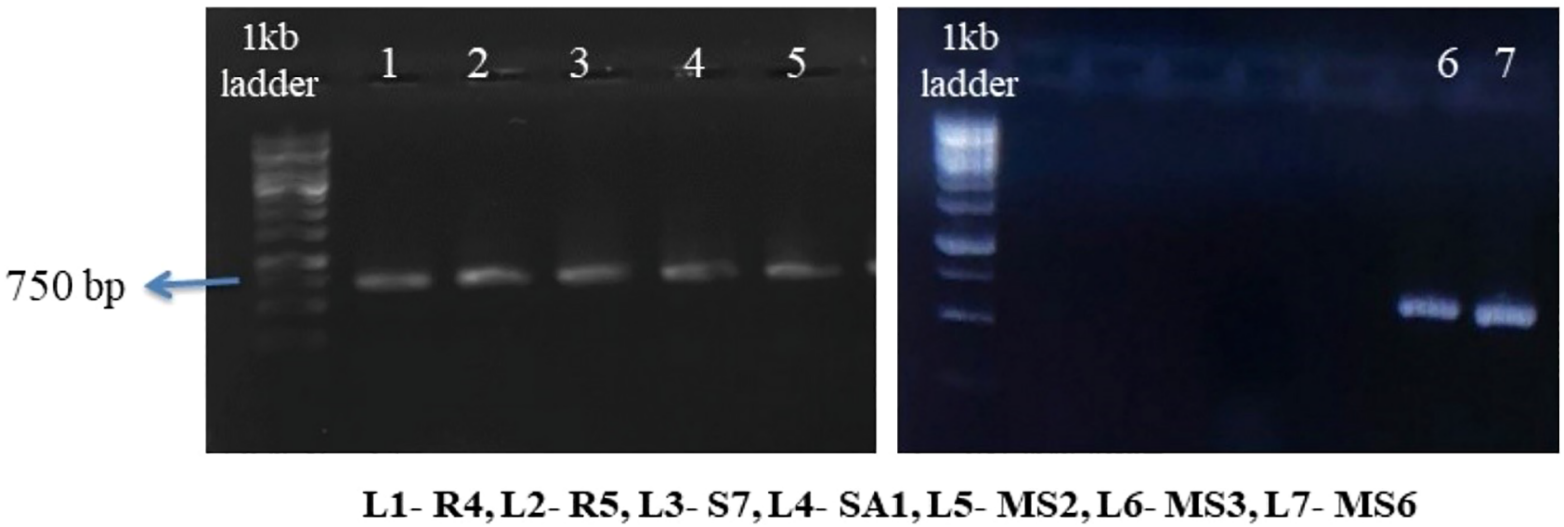
Amplification of isolates genomic DNA with 16sRNA primer showed sharp bands at 750bp.

The chosen bacterial strains underwent molecular characterization through 16S rRNA sequencing (**Figure 9**). The resulting sequences were submitted to the NCBI GenBank BLAST (Basic Local Alignment Search Tool) Nucleotide database. Sequences were submitted to the NCBI GenBank database with the following accession numbers (**Table 4**): R4-*Enterobacter hormaechei*(OP962461), R5-*Bacillus siamensis* (PP708989), S7-Pantoea agglomerans (OP962463), SA1-*Staphylococcus gallinarum* (OP962464), MS2-*Bacillus amyloliquefaciens*(OP954871), MS3-*Bacillus velezensis*(PP702344), and MS6-*K. Sacchari* (PP702289).

**Table 4:** Identification of selected plant growth-promoting rhizospheric bacteria from millets.

Bacterial isolates	Identified organism	Accession no
R4	*Enterobacter hormaechei*	OP962461
R5	*Bacillus siamensis*	PP708989
S7	*Pantoea agglomerans*	OP962463
SA1	*Staphylococcus gallinarum*	OP962464
MS2	*Bacillus amyloliquefaciens*	OP954871
MS3	*Bacillus velezensis*	PP702344
MS6	*Kosakonia sacchari*	PP702289

### Effect of isolates on seed germination

**Figure 10:**
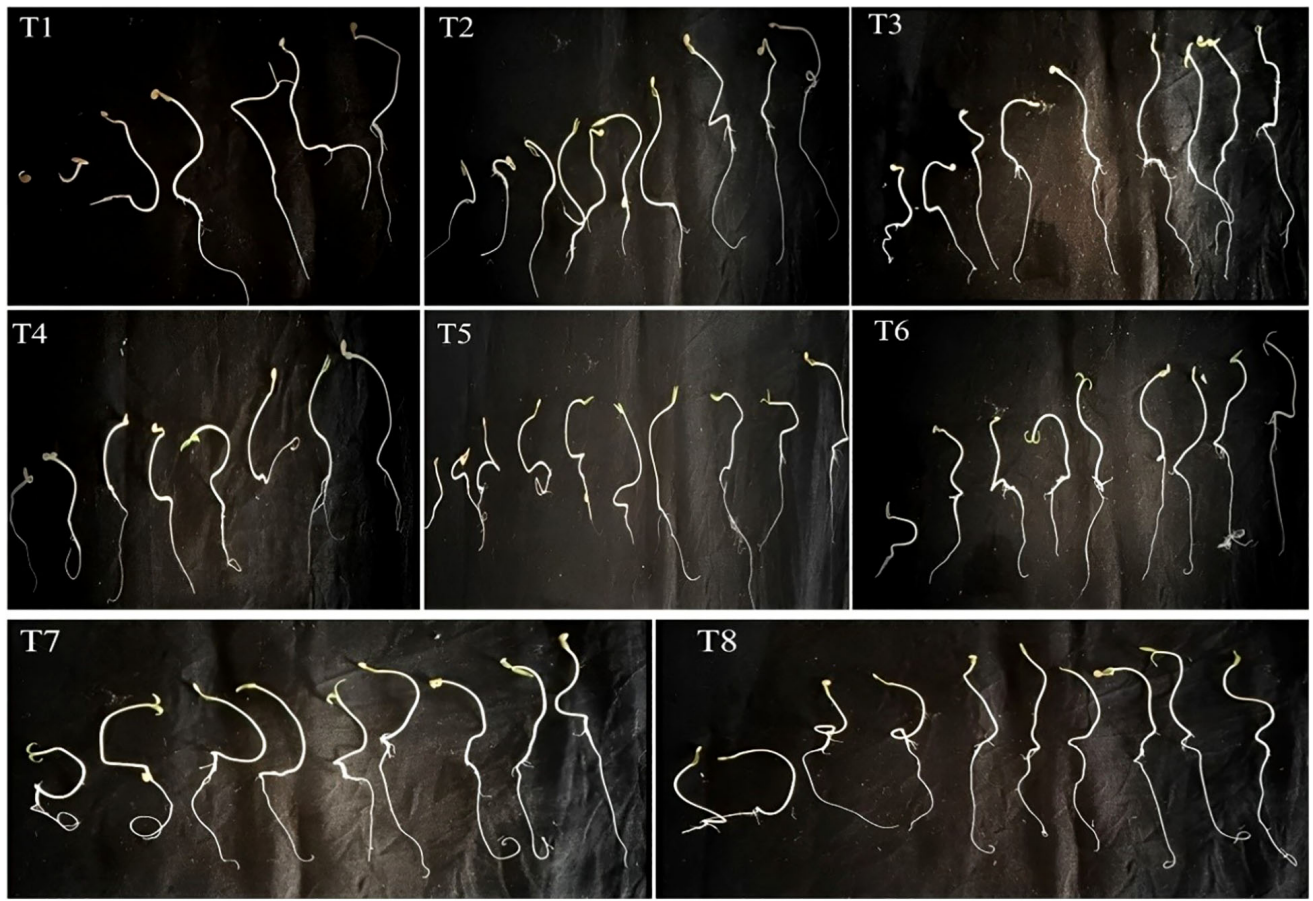
Ten-day-old Solanum lycopersicum treated with bacterial isolates. T1) Control, T2) Seeds treated with Enterobacter hormaechei, T3) *Bacillus siamensis*, T4) Pantoea agglomerans, T5) Staphylococcus gallinarum, T6) Bacillus amyloliquefaciens*, T*7) *Bacillus velezensis*, T8) Kosakonia sacchari respectively.

**Figure 11:**
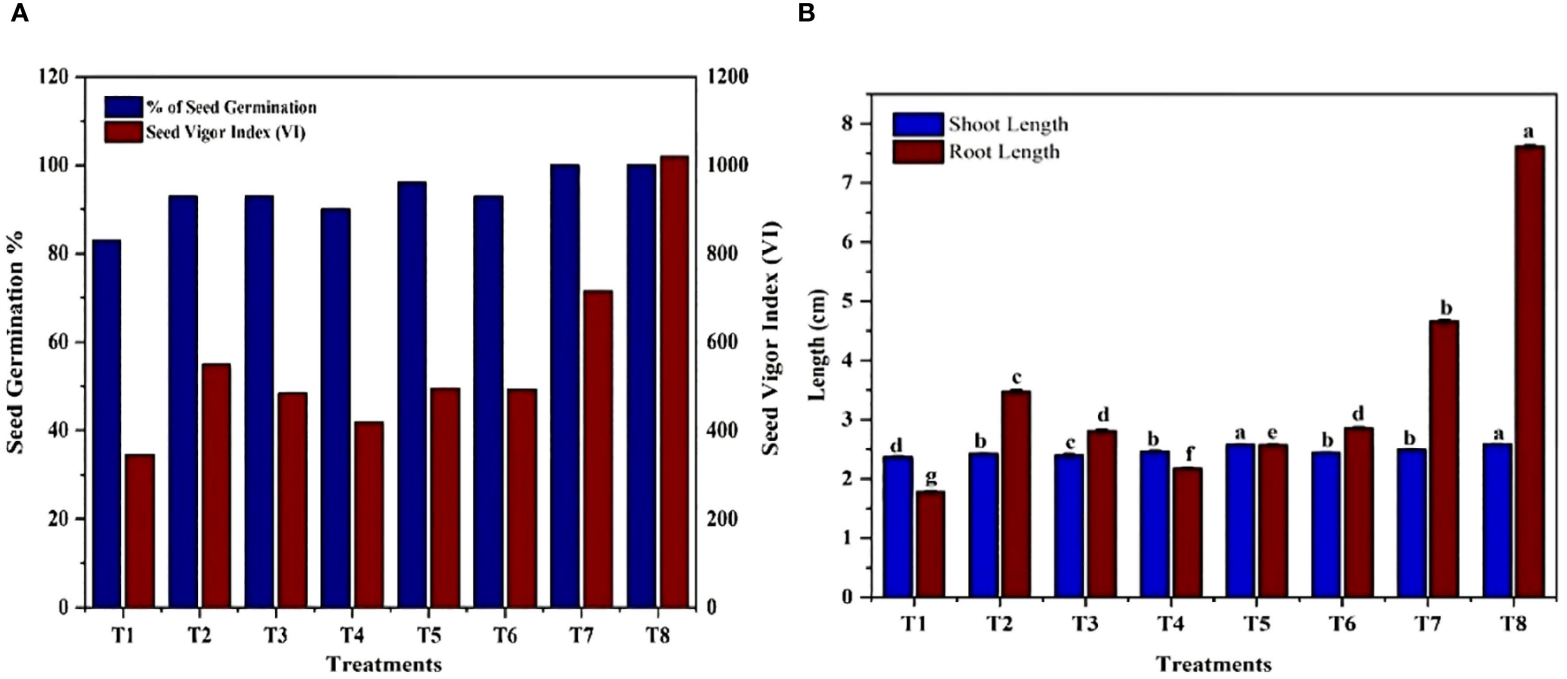
Impact of bacterial isolates on *Solanum lycopersicum* **(A)** seed germination percentage and seed vigor index. T1) Control, T2) Seeds treated with *Enterobacter hormaechei*, T3) *Bacillus siamensis*, T4) *Pantoea agglomerans*, T5) *Staphylococcus gallinarum*, T6) *Bacillus amyloliquefaciens, T7) Bacillus velezensis*, T8) *Kosakonia sacchari* respectively. **(B)** Shoot length and root length. T1) Control, T2) Seeds treated with *Enterobacter hormaechei*, T3) *Bacillus siamensis*, T4) *Pantoea agglomerans*, T5) *Staphylococcus gallinarum*, T6) *Bacillus amyloliquefaciens*, T7) *Bacillus velezensis*, T8) *Kosakonia sacchari* respectively.

The impact of isolated bacteria on the germination of *Solanum lycopersicum*(tomato) seeds was noted after 10 days of sowing. The findings of the current study indicate that seeds treated with PGPB exhibited significantly enhanced seed germination, seed vigor index, root length, and shoot length of the plantlets when compared to the control (**Figure 10**). The highest germination percentage and seed vigor index (1019), shoot length (2.58 cm), and root length (7.61cm) were recorded in *K. sacchari* (MS6), which is a classified genus from the family *Enterobacter* (Gu et al., 2014) this *Enterobacter sacchari* was reclassified as *K. sacchari* due to its association with sugarcane as a nitrogen fixer. To date, in India, there have been only two reports published on the isolation of *K. sacchari* from soil samples and for its PGP activities (Giri, 2019; Shahid et al., 2021). Following this, MS3 *has the seed vigor index of 715*, shoot length 2.49 cm, and root length 4.66 cm and in contrast, the control exhibited a germination percentage of 83%, seed vigor index of 344.45, shoot length of 2.37 cm, and root length of 1.78 cm (**Table 5**; **Figures 11A, B**). The results of the treatment were found to be significantly greater than those of the control.

**Table 5:** Impact of bacterial isolates on *Solanum lycopersicum* seed germination.

Isolates	Treatment	G %	SL (cm)	RL (cm)	VI
Control	T1	83%	2.37 ± 0.02	1.78 ± 0.02	344.45
R4	T2	93%	2.42 ± 0.01	3.48 ± 0.03	548.7
R5	T3	93%	2.40 ± 0.03	2.81 ± 0.03	484.53
S7	T4	90%	2.46 ± 0.02	2.18 ± 0.02	417.6
SA1	T5	96%	2.58 ± 0.01	2.57 ± 0.02	494.4
MS2	T6	93%	2.44 ± 0.008	2.86 ± 0.02	492.9
MS3	T7	100%	2.49 ± 0.01	4.66 ± 0.03	715
MS6	T8	100%	2.58 ± 0.01	7.61 ± 0.03	1019

G%, Germination percentage; SL, shoot length; RL, root length; VI, seed vigor index.

### Statistical analysis

**Figure 12:**
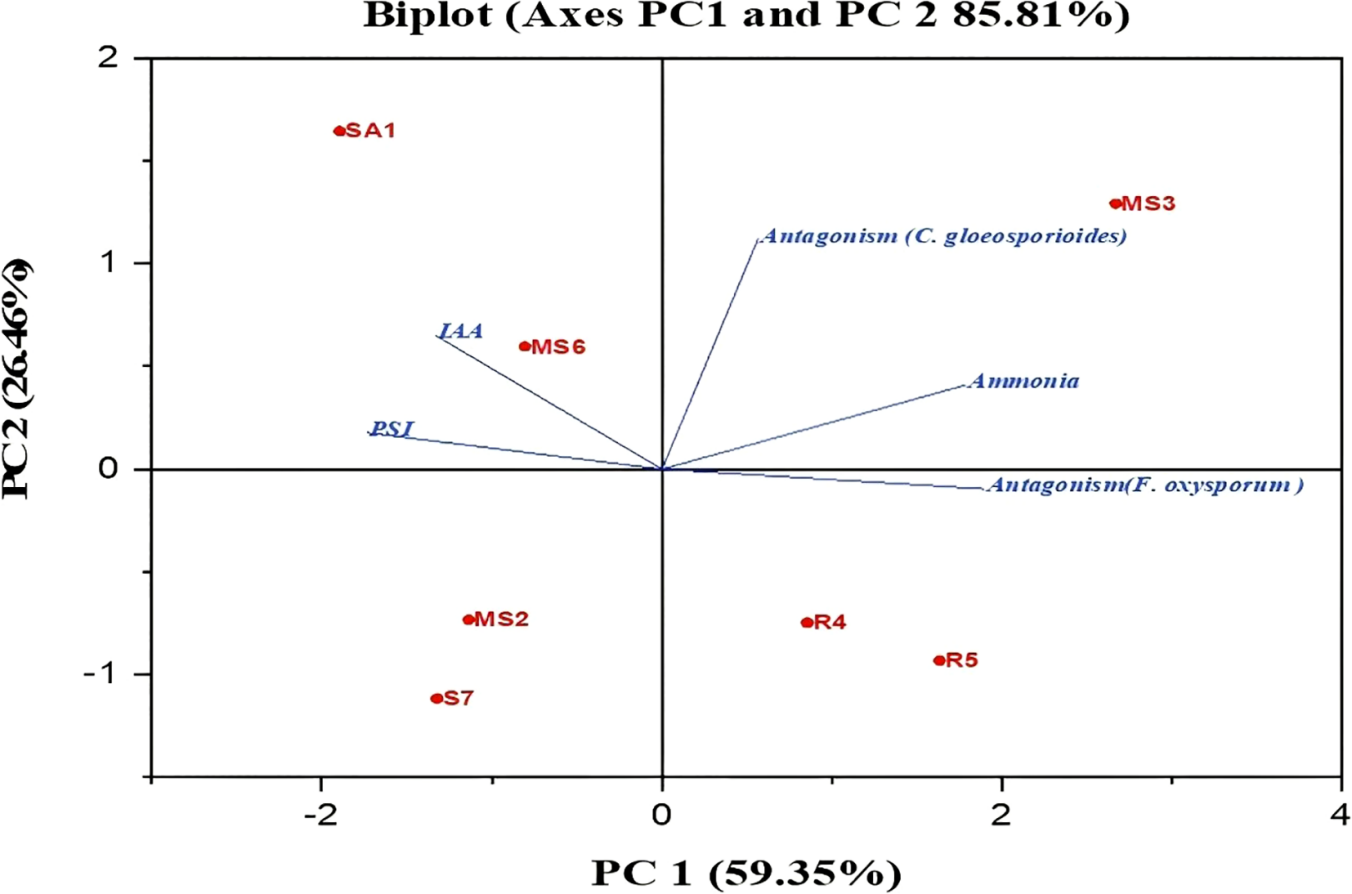
Principal component analysis (PCA) between the isolated bacterial stains and their PGP activity.

The plant growth-promoting potential of the bacterial isolates was evaluated based on their ability to produce IAA, ammonia, antagonistic activity, and phosphate solubilization. To understand how these traits were related, Principal Component Analysis (PCA) was carried out (**Figure 12**). The first two principal components explained most of the variation among the isolates, with PC1 showing an eigenvalue of 59.35% and PC2 with 26.46%. Together, they accounted for 85.81% of the overall variability. PC1 was mainly influenced by IAA production and phosphate solubilization, and PC2 by ammonia production and antagonistic activity. These results suggest that different isolates promote plant growth through a distinct combination of mechanisms, with some relying more on nutrient mobilization, such as IAA production and phosphate solubilization, and the others contributing through pathogen suppression and ammonia release.

## Discussion

In the rhizosphere, PGPRs reside on the root surface, promoting plant growth and development through both direct and indirect mechanisms (Meena et al., 2017). These mechanisms include nitrogen fixation, growth hormone production (auxins, gibberellins, cytokinins, etc.), ammonia production, inorganic phosphate solubilization, and the suppression of the growth of antagonistic microorganisms (Gerhardt et al., 2009). In the present work, 53 morphologically distinct isolates were isolated from millet rhizosphere soil using the spread plate technique. All the isolates were assessed for their nitrogen fixation ability, and 26 were found to be capable of fixing nitrogen when inoculated in a nitrogen-free medium. Nitrogen is a crucial element for the growth of the plant and yield; however, plants are unable to use nitrogen directly. The advantageous microorganisms found in the rhizosphere region are capable of converting atmospheric nitrogen into a usable form, namely ammonia, which is a major component of nucleic acids, amino acids, chlorophyll, and ATP (Andy et al., 2020). Similar results were reported by Reang et al. (2022) and Kanimozhi and Panneerselvam (2010) using the same medium, demonstrated that the isolates belonging to the genus *Halomonas* and *Azospirillum*, respectively, possess the ability to fix soil atmospheric nitrogen

It has been established that the synthesis of auxin by PGP bacteria (PGPB) represents the primary mechanism through which these bacteria can improve the growth of plants. In particular, the majority of the scientific literature focuses on IAA, the most prevalent biologically active auxin, which plays a pivotal role in plant growth by enhancing root and shoot development, elongation of roots and stems differentiation of vascular tissues, cell expansion, loosening of root cell walls, root bacterial colonization, stimulation of cell division, and defense against pathogens (Orozco-Mosqueda et al., 2023). In the present study, 14 of the 26 bacterial isolates produced IAA at varying concentrations; the highest IAA production of 0.37mg/mL was observed in SA1, and the lowest was in R5 (0.05 mg/mL). A comparable outcome was noted in the research conducted by the authors (Walpola and Arunakumara, 2016), they documented the maximum IAA production of 240 µg/mL and 332 µg/mL by *Enterobacter ludwigii* and *Enterobacter hormaechei*, respectively.

Ammonia production is a significant factor associated with the PGPB. Plants utilize the released ammonia from the PGPB as a nitrogen source, and the soil with abundant ammonia becomes alkaline, which prevents the growth of some fungi (Jha et al., 2012). In the current work, 23 bacterial isolates demonstrated positive results for ammonia production. Several reports exist on the production of ammonia in various concentrations by the genus Bacillus, particularly *B. licheniformis*, reported to produce nearly 4 µmol/mL (Goswami et al., 2014).

Phosphorus is a pivotal element that holds a crucial role in plant nutrition and numerous metabolic functions, including photosynthesis, respiration, energy transmission, signal transduction, and the biosynthesis of macromolecules (Anand et al., 2016). The majority of phosphorus, typically ranging from 95 to 99%, is present in an insoluble form that is not directly usable by plants. This can be achieved through the use of PGPB, which can lower the rhizosphere pH by secreting organic acids, including carboxylic acid and succinic acid. These acids bind to the phosphate group, forming calcium phosphate, thereby converting the insoluble phosphate to an available form (Sharma et al., 2013). In the present work, 15 isolates exhibited positive results with varying PSI values ranging from 2.36 to 3.80. The highest PSI was observed in *P. agglomerans* with a value of 3.80, and a similar result, with a 0.3 PSI difference, was reported in the research conducted by Prasad et al. (2022).

HCN is a volatile compound, a secondary metabolite, that inhibits the growth of phytopathogens (Kumar et al., 2014). Additionally, HCN production plays a pivotal role in root and shoot elongation, biomass production, and nitrogen accumulation (Marques et al., 2010). In the present work, only three isolates, namely R4 (*E. hormaechei*), R5 (*B. siamensis*), and MS3 (*B. velezensis*), demonstrated the capacity to produce HCN. Katiyar et al. (2017) documented the production of HCN by *E. hormaechei*, while Kanchiswamy et al. (2015) noted the production of HCN by the genus *Bacillus*.

The application of PGPB with siderophore-producing potential has been demonstrated to enhance plant growth by boosting iron intake, while simultaneously inhibiting phytopathogen growth, particularly fungi, by limiting iron availability (Kumari et al., 2018). Furthermore, the production of siderophore enables plants to take iron even in the presence of other metals like nickel and cadmium (Beneduzi et al., 2012). In the current work, 5 bacterial isolates exhibited positive results for siderophore production, namely R4 *(E. hormaechei)*, S7 (*P. agglomerans*), SA1 (*S. gallinarum*), MS3 (*B. velezensis*), and MS6 (*K. sacchari*). The different species from the genus *Enterobacte*r and *Pseudomonas* are reported by Alexander and Zuberer (1991) to produce siderophores in varying concentrations. Bhattacharyya et al. (2020) reported comparable results for the production of siderophore in varying concentrations from 99.26% to 52.31 % by various microorganisms belonging to the genus *Bacillus, Arthrobacter*, *Micrococcus, Pseudomonas, Staphylococcus, Ochrobactrum*, and *Exiguobacterium*. Shariati et al. (2017) reported that TonB and fepA are genes that encode for siderophore receptors in the bacteria *P. agglomerans*

Hydrolytic enzymes, such as protease, amylase, chitinase, lipase, and cellulase, act as defenders to protect plants from diseases by degrading the cell wall of the plant’s pathogenic microorganisms (Zerihun et al., 2019). PGPB with the potential to produce one or more of these lytic enzymes can exhibit biocontrol against plant diseases caused by harmful phytopathogenic bacteria and fungi (Rasool et al., 2021). Sritongon et al. (2023) demonstrated a comparable result, that the PGPR *Bacillus subtilis* produced significant production of amylase and protease. The enzyme chitinases strengthen plant defense by targeting chitin, the primary structural component of fungal cell walls, thereby inactivating pathogenic fungi without harming the host plant. In addition to enhancing resistance, these enzymes also contribute to improved plant growth and yield (Kumar et al., 2018). The genus *Serratia* and *Halomonas* are reported to produce a range of hydrolytic enzymes, cellulases, amylases, lipases, proteases, ureases, and chitinases (Ouali et al., 2024).

The antagonistic activity of the PGPB is an important feature, as it can be used as a biocontrol agent. By producing hydrolytic enzymes, siderophores, antibiotics, and inducing systemic resistance in plants, the PGPB can inhibit the growth of phytopathogens. Several studies have demonstrated the potential of PGPB isolates from the genera *Bacillus* and *Pseudomonas*, particularly *B. velezensis* and *P. fluorescens*, as effective biocontrol agents (Arkhipov et al., 2023).

The application of PGPB enhances seed germination due to the release of phytohormones, which play a crucial role in cell division (Pérez-García et al., 2023). PGPB produces phytohormones such as IAA, gibberellins, and cytokinins, which stimulate cell division and elongation during germination. Similar results for seed germination enhancement by PGPB have been reported in numerous vegetables, including *Daucus carota, Allium cepa, Capsicum annuum*, and *Solanum lycopersicum* (Lobato Ureche et al., 2021; Miljaković et al., 2022; Monalisa and Roy, 2022). The biplot PCA demonstrated a positive correlation between the IAA production and PSI. The results are comparable to those previously reported by the authors Barbaccia et al. (2022) and Sahin et al. (2004), respectively. The results demonstrated that the seven PGPBs have exhibited PGP characteristics, such as nitrogen fixation, ammonia and IAA production, phosphate solubilization, HCN production, siderophore production, hydrolytic enzyme secretion, and antagonistic activity. Further field investigations of these PGPBs will reveal their impact on PGP, the mechanism of action, and demonstrate their potential contribution to sustainable agriculture.

## Conclusion

This study offers valuable insights into plant growth-promoting rhizobacteria isolated from the millet rhizosphere, highlighting their significant role in enhancing tomato seed germination. The isolates exhibited multiple beneficial PGP traits, including nitrogen fixation, IAA production, ammonia production, phosphate solubilization, HCN production, siderophore production, hydrolytic enzyme production, and antagonistic activity, reflecting their potential in supporting plant growth and development. By harnessing the native microbial diversity of the millet rhizosphere, an underexplored but rich reservoir of agriculturally beneficial microbes, this present work highlights a sustainable approach to improve crop growth and development while reducing dependence on chemical fertilizers. Further investigation of the impact of these isolates on PGP will help to uncover the molecular mechanisms underlying their plant growth-promoting effects and assess their adaptability to diverse environmental conditions. Future work will prioritize optimization of bioformulation, consortia development, and field trials to develop targeted, crop-specific microbial inoculants capable of minimizing the usage of chemical fertilizers, enhancing soil fertility, and improving sustainable agriculture practices.

## Data Availability

Sequences were submitted to the NCBI GenBank database with the following accession numbers (**Table 4**): R4-Enterobacter hormaechei (OP962461), R5-Bacillus siamensis (PP708989), S7-Pantoea agglomerans (OP962463), SA1-Staphylococcus gallinarum (OP962464), MS2-Bacillus amyloliquefaciens (OP954871), MS3-Bacillus velezensis (PP702344), and MS6-K. Sacchari (PP702289).
